# Exploring the Mechanism on the Medullary Visceral Zone Inhibiting the Cholinergic Anti-inflammatory Pathway Induced by Sepsis

**DOI:** 10.1155/2020/1320278

**Published:** 2020-09-29

**Authors:** H. B. Li, Y. Zhou, A. H. Zhao, L. L. Guo

**Affiliations:** ^1^Emergency Department of the First People's Hospital of Guiyang of Guizhou Province, 550002, China; ^2^Department of General Surgery of Ezhou Central Hospital of Hubei Province, 436000, China; ^3^Surgery Department of the Third Hospital of Ezhou of Hubei Province, 436000, China; ^4^Pathology Department of the First People's Hospital of Guiyang of Guizhou Province, 550002, China

## Abstract

Inflammatory storm is an important pathological mechanism of multiple organ dysfunction, and it is associated with most deaths in septic patients, deserving to be studied. Recent findings have confirmed that the Medullary Visceral Zone (MVZ) regulates inflammation and immunity through the cholinergic anti-inflammatory pathway (CAP), but how sepsis affects the MVZ and leads to uncontrolled inflammation remain unclear. The current study reported that sepsis induced MVZ to inhibit CAP which underlies the inflammation storm. Our studies have shown that the rat models of sepsis prepared by cecal ligation and puncture had a higher inflammatory level, higher mortality, and higher Murine Sepsis Score. In septic rats, some indicators of heart rate variability (HRV) such as SDNN, HF band, RMSSD, SD1, and SD2 significantly reduced. In MVZ of septic rats, many cholinergic and catecholaminergic neurons showed apoptotic, with low expressions of tyrosine hydroxylase and choline acetyltransferase. The *α*7nAChR agonist GTS-21 can improve these pathologies, while the *α*7nAChR antagonist MLA is the opposite. Our study demonstrates for the first time that cholinergic and catecholaminergic neurons in MVZ went through significant apoptosis and inactiveness in sepsis, which contributes to the inhibition of CAP and acceleration of the inflammation storm in early sepsis. Intervening with CAP has a significant effect on the activity and apoptosis of MVZ neurons while altering systemic inflammation and immunity; in addition, for the first time, we confirmed that some indicators of HRV such as SDNN, HF band, RMSSD, SD1, and SD2 can reflect the activity of CAP, but the CAP interference had little effect on these indicators.

## 1. Introduction

In 2017, approximately 48.9 million patients were diagnosed with sepsis worldwide which caused approximately 1.1 million deaths, accounting for 19.7% of all-cause mortality [1]. Sepsis is an abnormal response of the host to infection, which can lead to life-threatening organ dysfunction. The immune system is activated in response to infection through pathogen-associated molecular patterns, involving Toll-like receptors on the cells of the innate immune system [2]. This interaction triggers the release of both proinflammatory and anti-inflammatory mediators such as tumor necrosis factor-*α*, interleukin 1, interleukin 2, interleukin 6, and interleukin 8, which cause neutrophil-endothelial cell adhesion, activate the complement and clotting cascades, and contribute to multiple organ dysfunction and even death [3]. Early inflammation and immune disorders in infectious disease are critical to the formation and progression of sepsis; even the survival of patients with sepsis also depends on the intensity of inflammation and the balance between proinflammatory and anti-inflammatory strength [4].

A series of clinical and experimental studies conducted in recent years have revealed that the Medullary Visceral Zone (MVZ) regulates inflammation and immunity dynamically through the cholinergic anti-inflammatory pathway (CAP) [5]. Therefore, the uncontrolled inflammation and immunity in sepsis will inevitably be related to the disorder of MVZ regulation. Studies have shown that systemic inflammation can induce MVZ neuroinflammation [6, 7] in many ways, such as through the leak of cytokines from circumventricular organs neighboring the blood-brain barrier (BBB) [8], or saturable diffusion of cytokines through the BBB [9], or paracrine secretion of cytokines from the brain endothelial cells [10], or the inflammation signals transmitted to the brain through the vagus nerve [11]. Neuroinflammation results in CAP dysfunction which may be a key cause of the septic inflammatory storm, but the pathologies of MVZ under neuroinflammation and its impact on CAP remain unclear. This study attempts to use agonists and antagonists to simulate and antagonize the function of CAP in sepsis rats to study the apoptosis and activity of cholinergic neurons and catecholaminergic neurons in MVZ and simultaneously study the regulatory activity of CAP and the intensity of systemic inflammation and immunity, through exploring the pathologies of MVZ in sepsis and its effect on the regulation of CAP to provide ideas for the treatment of sepsis.

## 2. Methods

### 2.1. Animals

64 adult, male Sprague-Dawley rats (8 weeks old, 250-280 g, License Number: SYXK (Hubei)2018-0104) were purchased from the Experimental Animal Center of Three Gorges University (Sale License Number: SCXK(Hubei)2017-0012). Rats were housed in the Guizhou Medical University Experimental Animal Center and maintained under temperature control (21 ± 0.5°C) and a 12 h light-dark cycle (lights on during 05:00–17:00). Standard chow and tap water were provided ad libitum. Animals were acclimatized for 7 days before experimental manipulations. All experimental procedures were carried out in compliance with the guidelines of the institutional animal care and use committee (IACUC) of the First Hospital of Guiyang (IACUC number 20190107).

### 2.2. Animal Grouping and Treatment

After 7 days of adaptive feeding, rats were divided into three groups according to the random number table: (1) control group: rats (*n* = 8) were fed as usual without any treatment; (2) sham group: rats (*n* = 8) were subjected to open and suture of the abdominal cavity without cecal ligation and puncture (CLP) preparing for sepsis models [12]; afterwards, they were accepted intraperitoneal injection of piperacillin (50 mg/kg, i.p. tid×3 d); (3) sepsis group: the sepsis rats (*n* = 48) were prepared with the CLP method; the operation process is as follows: the abdominal cavity was cut about 2 cm along the white line of the abdomen to find the cecum, about 1/3 of the cecum was ligated with a 5-0 suture, and the ligated cecum was pierced with a 21G needle twice. A small amount of intestinal contents from the puncture hole were gently squeezed, the processed cecum was returned into the abdominal cavity, the inner layer was sutured with 5-0 sutures, and the outer layer was sutured with 3-0 sutures. The rats were under anesthesia with isoflurane inhalation during all the procedures. The rats in the sepsis group were delayed to recover to their consciousness, the bodies were curled up and inactive, and they breathed fast and had little and slow response to the outside world. These appearances indicated that the model is successful. One hour after waking up, they were randomly divided into 3 groups, 16 rats in each group: (a) model group: accepting intraperitoneal injection of piperacillin (50 mg/kg, i.p. tid×3 d) and saline (1 mL/100 g, i.p. tid×3 d); (b) GTS-21 group, besides piperacillin used in the model group, intraperitoneal injection of GTS-21 (a selective *α*7 nicotinic acetylcholine receptor agonist; it can mimic CAP in acting on the *α*7 nicotinic acetylcholine receptors on monocytes and neutrophils to downregulate NF-*κ*B and inhibit the transcription and release of inflammatory factors, produced by MCE; Lot: 29834. Dosage and duration: 4 mg/kg, i.p. tid×3 d) was given to each rat [13]; (c) methyllycaconitine (MLA) group: besides piperacillin used in the model group, intraperitoneal injection of MLA (a powerful and selective nicotine acetylcholine receptor antagonist; the terminals of CAP release acetylcholine, so it can simulate to block CAP's effect on immune cells and aggravate inflammation, produced by MCE; Lot: HY-N2332A/CS-0021211. Dosage and duration: 4.8 mg/kg, i.p. tid×3 d) was given to each rat [14]. After 3 days, the rats were sacrificed to collect blood and medullary tissue for analysis under anesthesia with isoflurane inhalation.

### 2.3. Murine Sepsis Score (MSS) and Rat Mortality Assessment

MSS was used to assess the severity of sepsis [15]. The scoring rules are shown in Table 1. The higher the score, the more severe the rat. Three experimenters evaluated each rat and get a score separately, and the average score was used to judge the severity of sepsis of each rat. In addition, the mortalities of each group were documented at 3 consecutive days after the model was built.

### 2.4. HRV Monitoring

Six hours after the CLP operation, the HRV monitoring for all rats began [16]. The BL-420F Biological Experiment System was used to record the rats' electrocardiogram on lead II for 5 minutes. The anesthetized rat was taken with isoflurane inhalation in the supine position; the electrode and rat limbs were sterilized with 75% alcohol, respectively; and the white, black, and red recording needles were inserted into the right forelimb, right hindlimb, and left hindlimb subcutaneously. The parameters were set as follows: sampling rate is 1 K/s and low-pass filter is 1 kHz. After electrocardiogram recording, HRV were performed with BL-420F Biosignal Acquisition and Analysis System supplied by Chendu Taimeng Software Co. The frequency domain, time domain, and nonlinear analysis of HRV were performed.

It was conducted at 10:00-11:00 AM every morning 2 hours after intraperitoneal injection at 8:00 AM for the following 2 days. The main indicators include the standard deviation of all NN intervals (SDNN), the root mean square successive difference of continuous RR interval (RMSSD), total power (TP), very low frequency (VLF), low frequency (LF, absolute or standardized unit; 0.20 to 0.75 Hz), high frequency (HF, absolute or standardized unit; 0.75 to 3.00 Hz), standard deviation of width of a scatterplot of successive pairs of RR intervals (SD1), and standard deviation of length of a scatterplot of successive pairs of RR intervals (SD2).

### 2.5. Enzyme-Linked Immunosorbent Assay (ELISA)

On the 3rd day, after HRV monitoring, 6 rats from each group were anesthetized with isoflurane inhalation [17, 18]; then, the chest cavity was dissected, and 2 mL of blood was taken from the right atrium for ELISA and 2 mL for flow cytometry (FCM). For ELISA, blood was preserved at room temperature for 2 h prior to centrifugation at 3000 r/min at 4°C for 10 min; then, the upper layer of the serum was separated and stored in the refrigerator at -80°C for detection. For FCM, fresh blood was taken for testing. According to the manufacturer's instructions [19], the serum levels of TNF-*α*, IL-1*α*, IL-10, IL-6, HMGB1, and sCD14 were determined with ELISA.

The main related antibodies include: rat anti-TNF-*α* (lot number: E-EL-R0019c, manufacturer: Elabscience), rat anti-IL-1*α* (lot number: E-EL-R0011c, manufacturer: Elabscience), rat anti-IL-6 (lot number: E-EL-R0015c, manufacturer: Elabscience), rat anti-IL-10 (lot number: E-EL-R0016c, manufacturer: Elabscience), rat anti-HMGB-1 (lot number: E-EL-R0505c, manufacturer: Elabscience), and rat anti-sCD14 (lot number: CSB-E11178r, manufacturer: Cusabio).

### 2.6. Flow Cytometry (FCM) Analysis

According to the FCM kit's instructions, the lymphocyte surface antigen was labeled by a double fluorescent antibody, TH17 lymphocytes were labeled with CD4-FITC (fluorescein isothiocyanate)/IL-17A-PE (phycoerythrin), and Treg lymphocytes were labeled with CD4-FITC/CD25-PE. The procedures are as follows: 2 mL fresh collected blood was centrifuged at 400-500g at room temperature for 30 minutes to acquire leukocytes; after repeated washing and centrifuging, 0.1 mL cell suspension was incubated and fluorescent-labeled antibody was added to incubate again; eventually, the cells were resuspended in 0.2 mL PBS containing 0.5% BSA and analyzed by flow cytometry. The antigen-labeled monoclonal antibodies used include CD25-PE monoclonal antibody (manufacturer: Invitrogen, lot number: 12-0390-82), CD4-FITC monoclonal antibody (manufacturer: Invitrogen, lot number: 11-0040-82), and IL-17A-PE monoclonal antibody (manufacturer: Invitrogen, lot number: 12-7177-81).

### 2.7. Sampling and TdT-Mediated dUTP Nick-End Labeling (TUNEL)

After blood collection, rats were perfused transcardially with phosphate-buffered saline (PBS, 0.01 M, 150 mL at pH 7.4) followed by a paraformaldehyde (PFA, 350 mL) solution. Afterward, the whole brains were removed and fixed in 4% PFA for 3 h at 4°C. Fixed brains were preserved at 4°C with 0.01 M PBS containing 30% sucrose for 72 h. The medulla oblongata was acquired and conventionally embedded with paraffin to prepare sections (30 *μ*m). The paraffin sections from different groups were taken, dewaxed, and immerged in Proteinase K solution. Then, the TdT (Terminal Deoxynucleotidyl Transferase) buffer was added to incubate. At last, DAPI (Beyotime Biotechnology, lot number: C1002) and fluorescence quencher (SouthernBiotech, lot number: 0100-01) were added to stain the nuclei and the apoptotic cells. The sections were observed under a fluorescent microscope (Olympus BX53 biological microscope). The normal cell nuclei were stained blue, and the apoptotic cell nuclei were stained red. ImageJ software was used to calculate the apoptosis index [20]. 10 images from every group were randomly selected; the ratio of the number of apoptotic cells to the total number of cells in each image was calculated, and the average percentage of the apoptosis is the apoptosis index of the group.

### 2.8. Immunofluorescence

After dehydration, the sections were blocked with 10% normal goat serum (1 h) and then incubated for 24 h in a cocktail of primary antibodies for the labeling of Caspase3 (produced by Wuhan Sanyan Bio. Co., China. Lot: 66470-2-IG, dilution: 1 : 50), tyrosine hydroxylase (TH, produced by Wuhan Boster Co., China. Lot: BM4568, dilution: 1 : 50), or choline acetyltransferase (CHAT, produced by Wuhan Bioss Co., China. Lot: bs-2423R, dilution: 1 : 50). The reactions with primary antibodies were followed by 4 h of incubation in the presence of a fluorescent-labeled secondary antibody (FITC-labeled goat anti-rabbit IgG, produced by Wuhan Boster Co., China. Lot: BA1105, dilution: 1 : 100; Cy3-labeled goat anti-mice IgG, produced by Wuhan Boster Co., China. Lot: BA1031, dilution: 1 : 100). Finally, the sections were mounted on gelatin-coated slides and covered with mounting medium with DAPI, for nuclear staining of all cells present in the slice [21]. The images from the different experimental groups were captured with an Olympus BX53 Biological Microscope. The normal nuclei were stained blue, cholinergic neurons expressing CHAT and catecholaminergic neurons expressing TH were stained green, and apoptotic neurons expressing Caspase3 were stained red. Three images with 400-fold enlargement from every group were analyzed with imagepro (ipp6.0) software [22], and the average densities were acquired.

### 2.9. Statistics

Measurement data were expressed as the mean ± standard deviation ($\bar{X}\pm \text{SD}$). Data were statistically processed with SPSS 19.0 software package. Intergroup differences were analyzed with ANOVA. The Levene homogeneity test is performed first. The comparison of homogeneous data is determined by the Bonferroni test; otherwise, they were judged by the Tamhane test. The rats' mortality was analyzed with the Kaplan-Meier survival curve and *χ*^2^ test. *P* < 0.05 was considered statistically significant.

## 3. Results

### 3.1. Rat Mortality and MSS

**Figure 1:**
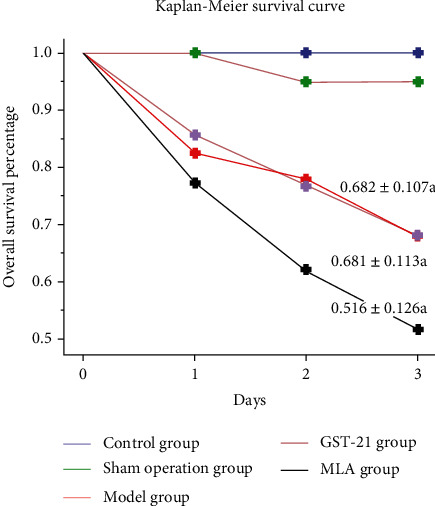
Kaplan-Meier survival curve among the five groups. On the first postoperative day, the model group, GTS-21 group, and MLA group had more deaths than the control group. On the second and third days, the cumulative survival rates of the GTS-21 group were close to the model group, and the cumulative survival rate of the MLA group continued to decline. There was no statistical difference in the cumulative survival rates of the three sepsis groups (*χ*^2^ = 1.21, *P* > 0.05), but they were significantly lower than that of the control group and the sham group. Both the control group and the sham group had no death, and there were significant differences between the five groups (*χ*^2^ = 14.21, *P* < 0.01). a: *P* < 0.05 vs. control group.

The mortality rates of the control group, sham group, model group, GTS-21 group, and MLA group were, respectively, 0%, 0%, 56.3%, 50%, and 68.8%. There was a significant difference in the survival rate among the five groups (*χ*^2^ = 14.210, *P* < 0.01); however, although the overall survival percentages of the three sepsis groups had no significant difference (*χ*^2^ = 1.21, *P* > 0.05), they were much lower than those in the control group and the sham group (Figure 1).

**Figure 2:**
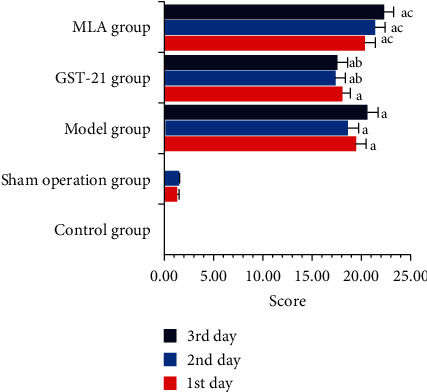
MSS of rats in different groups. The average MSS scores of the model group, GTS-21 group, and MLA group were much higher than those of rats in the control group and those of rats in the control group. *P* value and 95% CI (confidence interval) were, respectively, 0.000, -21.215∼-17.745; 0.00, -19.064∼-16.222; and 0.00, -23.359∼-18.841. The average MSS score of the MLA group was much higher than that of rats in the GTS-21 group; the *P* value and 95% CI were, respectively, 0.003 and 0.906∼6.009. The average MSS scores of the GTS-21 group were much lower than those of the model group; the *P* value and 95% CI were, respectively, 0.039 and -3.621∼-0.054. a: *P* < 0.05 vs. control group; b: *P* < 0.05 vs. model group; c: *P* < 0.05 vs. GTS-21 group.

In the same day, the MSS scores of the model group, GTS-21 group, and MLA group were significantly higher than that of the control group, and the MSS score of the MLA group was significantly higher than that of the GTS-21 group (*P* < 0.05, Figure 2). In addition, the scores of the same group on different days were not significantly different (see Figure 2).

### 3.2. Effects of Sepsis on Inflammatory Cytokine Levels and CAP Intervention

**Figure 3:**
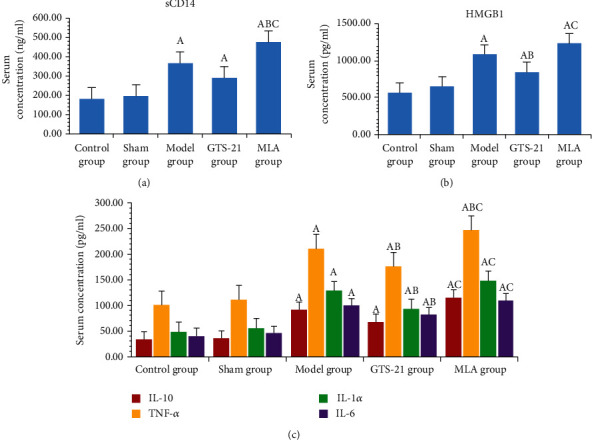
The concentration of serum proinflammatory cytokines among the groups of rats. sCD14: soluble CD14; precepsin; HMGB-1: high mobility group box-1; IL-10: interleukin 10; TNF-*α*: tumor necrosis factor alpha; IL-1*α*: interleukin 1 alpha; IL-6: interleukin 6. (a) The serum sCD14 concentration (ng/mL) among different groups; it is a marker of inflammation in the late stage of sepsis. (c) Serum concentration of IL-10, TNF-*α*, IL-1*α*, and IL-6 (pg/mL) among groups. The serum sCD14 levels of the model group, GTS-21 group, and MLA group were much higher than those of the control group. The *P* value and 95% CI were, respectively, 0.000, -286.858∼-79.4662; 0.44, -209.616∼-2.223; and 0.000, 400.647∼-193.255, and so were HMGB1, TNF-*α*, IL-10, and IL-6. For HMGB1, they were 0.000, -766.215∼-289.627; 0.012, -527.403∼-50.815; and 0.000, -917.862∼-441.275. For TNF-*α*, they were 0.000, -139.923∼-82.198; 0.000, -104.224∼-46.494; and 0.000, 175.466. For IL-1*α*, they were 0.000, -102.305∼-57.714; 0.000, -66.369∼-21.777; and 0.000, -121.697∼-77.106. For IL-10, they were 0.000, -83.572∼-32.005; 0.006, -59.758∼-8.191; and 0.000, -108.319∼-56.752. For IL-6, they were 0.000, -77.851∼-42.647; 0.000-59.996∼-24.792; and 0.000, -87.186∼-51.982. The serum sCD14, HMGB1, TNF-*α*, IL-1*α*, IL-10, and IL-6 levels of the MLA group were much higher than those of rats in the GTS-21 group; the *P* value and 95% CI were, respectively, 0.000, -294.727∼-87.3357; 0.001, 152.166∼628.754; 0.000, 42.107∼99.837; 0.000, 33.033∼77.624; 0.000, 22.777∼74.344; and 0.001, 9.588∼44.792. A: *P* < 0.05 vs. control group; B: *P* < 0.05 vs. model group; C: *P* < 0.05 vs. GTS-21 group.

Inflammatory cytokines (including TNF-*α*, IL-1*α*, IL-10, IL-6, HMGB1, and sCD14) in the model group, GTS-21 group, and MLA group were significantly higher than those in the control group and the sham group (*P* < 0.05). The serum concentration of TNF-*α*, IL-1*α*, IL-6, and HMGB1 in the GTS-21 group was significantly lower than those in the model group (*P* < 0.05). All inflammatory cytokine levels reached the highest in the MLA group, and there were significant differences when compared to the GST-21 groups (*P* < 0.05) (see Figure 3).

### 3.3. Effects of Sepsis on the Percentage of CD4+CD25+Treg and TH17 Lymphocyte and CAP Intervention

**Figure 4:**
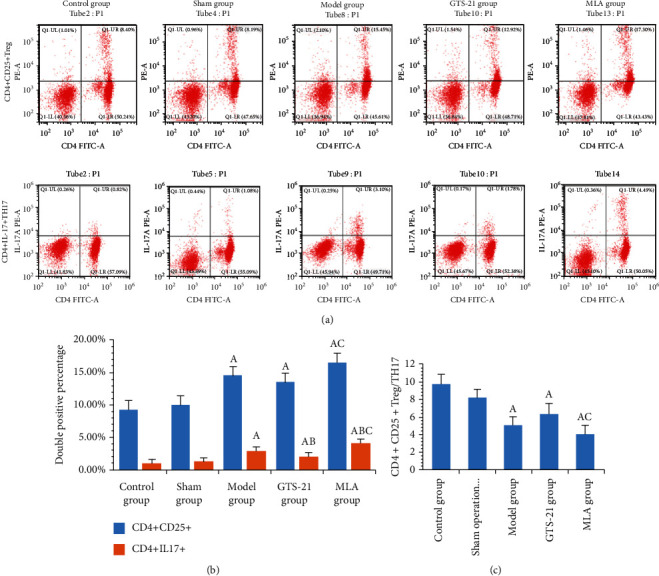
Study of the cholinergic anti-inflammatory pathway (CAP) on immune regulation. (a) Representative scatter plot images of flow cytometry of CD4+CD25+Treg and CD4+IL-17+TH17 lymphocyte among different groups. (b) Double positive percentage of CD4+CD25+Treg and CD4+IL-17+TH17 lymphocytes among different groups. (c) The ratio of CD4+CD25+Treg/TH17 among groups. CD4+CD25+Treg lymphocyte percentages of the model group, GTS-21 group, and MLA group were much higher than those of the control group. The *P* value and 95% CI were, respectively, 0.000, -7.893∼-2.754; 0.002, -6.773∼-1.634; and 0.000, -9.846∼-4.707, and so were CD4+IL-17+TH17 lymphocyte percentages; they were 0.000, -2.668∼-1.279; 0.002, -1.851∼-0.462; and 0.000, -3.858∼-2.469. CD4+CD25+Treg and CD4+IL-17+TH17 lymphocyte percentages of the MLA group were much higher than those of the GTS-21 group. The *P* value and 95% CI were, respectively, 0.018, 0.504∼5.643; 0.000, 1.312∼2.701. A: *P* < 0.05 vs. control group; B: *P* < 0.05 vs. model group; C: *P* < 0.05 vs. GTS-21 group.

The percentages of CD4+CD25+Treg and TH17 lymphocyte of rats in the model group, GTS-21 group, and MLA group were significantly higher than those in the control group and sham group (*P* < 0.05). The percentage of the TH17 lymphocyte in the GTS-21 group was much lower than that in the model group (*P* < 0.05). The percentages of Treg and TH17 lymphocytes in the MLA group were significantly higher than those in the GTS-21 group (*P* < 0.05). The ratio of Treg/TH17 in the model group was significantly lower than that in the control group (*P* < 0.05); it was not significantly higher in the GTS-21 group than that in the model group, while in the MLA group, it was significantly lower than that in the GTS-21 group (*P* < 0.05) (see Figure 4).

### 3.4. Results of HRV Analysis

Compared with the control group and the sham group, the time domain indicators (including SDNN and RMSSD), frequency indicators (HF), and nonlinear indicators (including SD1 and SD2) of HRV were significantly reduced in the model group, GTS-21 group, and MLA group (*P* < 0.05), but these indicators had no statistical difference among the three sepsis groups; there was no statistical difference when comparing the frequency index of HRV (including TP, VLF, LF, and LF/HF) and the nonlinear index SD1/SD2 among groups (see Table 2). In the further subgroup analysis, there was no statistical difference in the same HRV index among the three sepsis groups in the same monitoring day.

### 3.5. Effects of Sepsis on Apoptosis of MVZ Neurons and CAP Intervention

**Figure 5:**
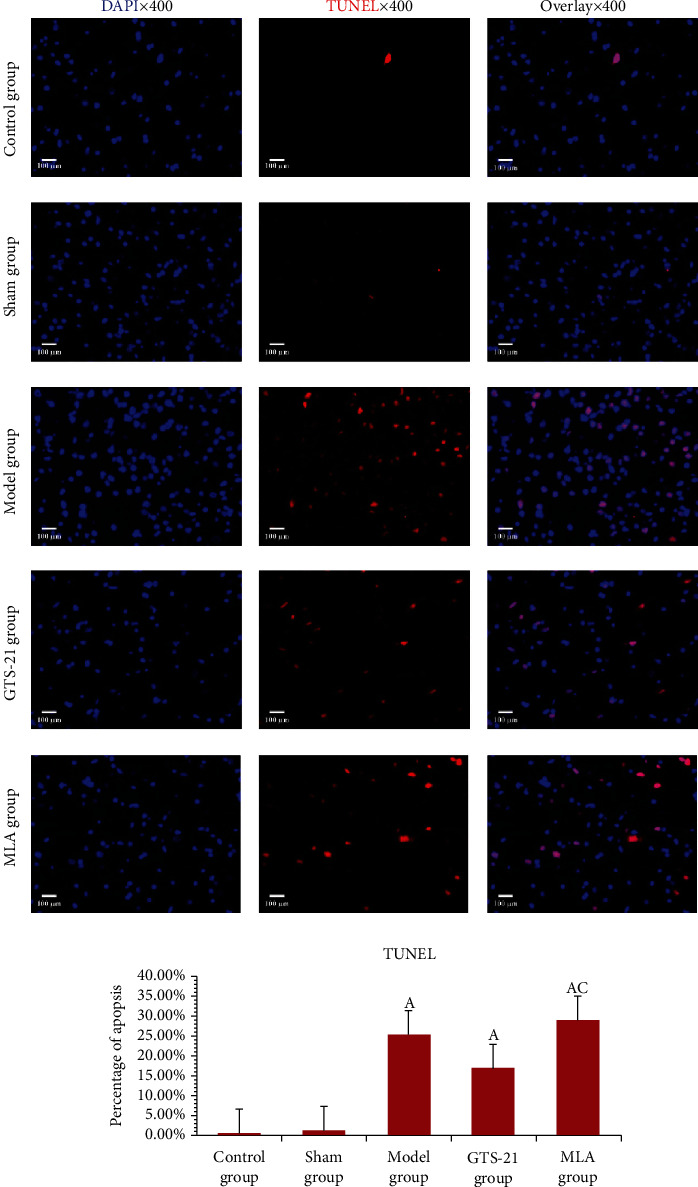
Cell apoptosis in MVZ among different groups. The results of TUNEL show that much more apoptotic percentages occurred in the sepsis groups (including the model group, GTS-21 group, and MLA group) than in the control group. The *P* value and 95% CI were, respectively, 0.000, -0.337~-0.160; 0.000, -0.253~-0.077; and 0.000, -0.374~-0.197. GTS-21 decreased the apoptotic percentage when compared with the model group (0.074, -0.00~0.172). MLA significantly increased the apoptotic percentage when compared to the GTS-21 group; it reached a significant difference (the *P* value and 95% CI were, respectively, 0.003 and -0.209~-0.032). The scale bars at the bottom left of the images represent 100 *μ*m. a: *P* < 0.05 vs. control group; c: *P* < 0.05 vs. GTS-21 group.

In the TdT-mediated dUTP Nick-End Labeling (TUNEL) experiment, there were more apoptotic MVZ cells in the sepsis groups than those in the control group (*P* < 0.05). When compared to the model group, there was a tendency for GTS-21 to inhibit apoptosis; on the contrary, MLA significantly increases the percentage of apoptotic cells; the percentage of apoptotic cells in the MLA group was significantly higher than that in the GTS-21 group (*P* < 0.05) (see Figure 5).

**Figure 6:**
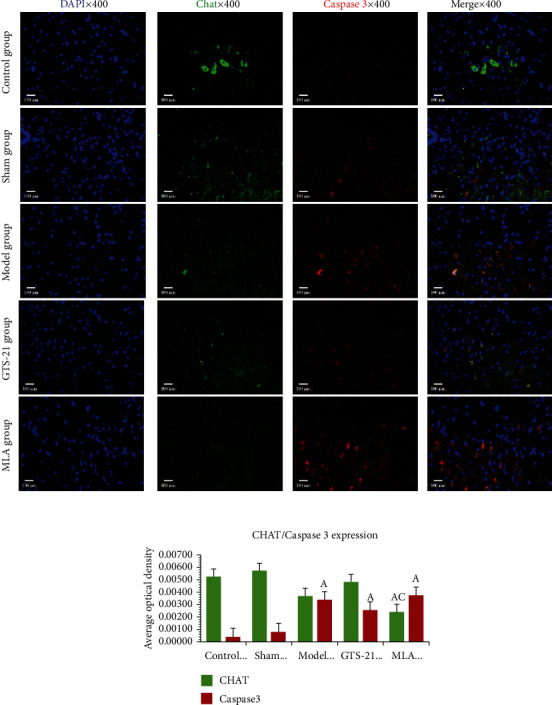
Cholinergic neuron apoptosis in MVZ among the different groups. The images mainly cover NST and VLRN of MVZ. DAPI: 4′,6-diamidino-2-phenylindole, a fluorescent blue dye capable of binding strongly to DNA. CHAT: choline acetyltransferase, used for labeling cholinergic neurons (stained green with FITC); Caspase3: indicator for apoptotic cell (stained red with Cy3). The scale bars at the bottom left of the images represent 100 *μ*m. a: *P* < 0.05 vs. control group; c: *P* < 0.05 vs. GTS-21 group.

The further immunofluorescence double-label colocalization studies found that the expression of CHAT in the model group had a tendency to downregulate when compared to the control group, and GTS-21 had a tendency to enhance it. There was no significant difference for the expressions of CHAT among the control group, sham group, model group, and GTS-21 group; the expression of CHAT in the MLA group was significantly lower when compared with the control group and GTS-21 group. The expression of Caspase3 in the sepsis groups significantly upregulated when compared with the control group (*P* < 0.05), but there was no statistical significance among the three sepsis groups (see Figure 6).

**Figure 7:**
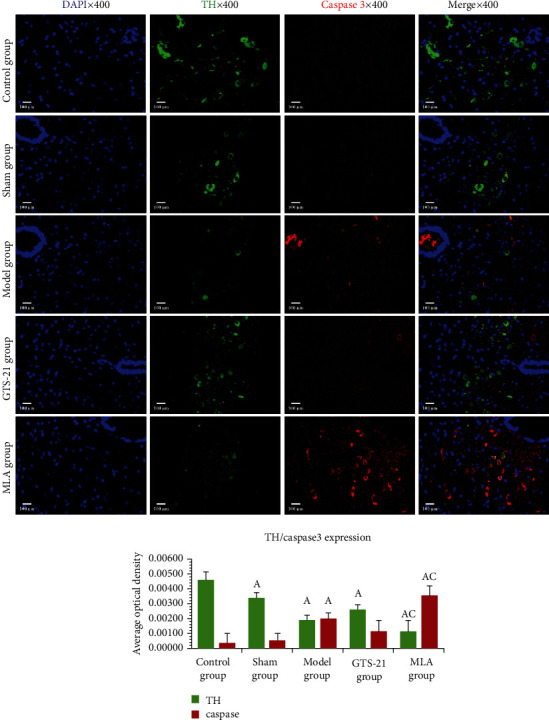
Catecholaminergic neuron apoptosis in MVZ among different groups. The images mainly cover the NST and VLRN of MVZ. DAPI: 4′,6-diamidino-2-phenylindole, a fluorescent blue dye capable of binding strongly to DNA. TH: tyrosine hydroxylase, used for labeling catecholaminergic neurons (stained green with FITC); Caspase3: indicator for apoptotic cell (stained red with Cy3). The scale bars at the bottom left of the images represent 100 *μ*m. a: *P* < 0.05 vs. control group; c: *P* < 0.05 vs. GTS-21 group.

In the TH/Caspase3 immunofluorescence colocalization study, TH expressions in the sham group, model group, GTS-21 group, and MLA group were significantly lower than those in the control group (*P* < 0.05); it was lowest in the MLA group. The expressions of Caspase3 in the model group and the MLA group were much higher than those in the control group (*P* < 0.05); GTS-21 had the tendency to suppress it. The expression of Caspase3 reached the highest in the MLA group; there was a statistical difference when it was compared between the MLA group and the GTS-21 group (*P* < 0.05) (see Figure 7).

### 3.6. Correlation Analysis between HRV Indexes and MSS, Inflammatory Cytokines, Immune Regulation, Expression of CHAT, and TH/Caspase3

Through Pearson correlation analysis, it was shown that SDNN, RMSSD, HF, SD1, and SD2 had a strong negative correlation with MSS scores and the expression of Caspase3 (*P* < 0.01 or 0.05); SDNN, RMSSD, SD1, and SD2 were strongly negatively correlated with TNF-*α*, IL-1*α*, IL-6, IL-10, sCD14, HMGB1, and CD4+CD25+Treg and TH17, whereas they were positively correlated with the expression of CHAT and TH (*P* < 0.01 or *P* < 0.05). Though HF was negatively correlated with all the measured cytokines (*P* < 0.05), it was not significantly correlated with the percentage of the immune-regulating lymphocytes; LF/HF was positively correlated with sCD14 (*P* < 0.05); Treg/TH17 was positively correlated with SDNN, RMSSD, SD1, and SD2 (*P* < 0.01 or 0.05). There was no significant correlation between the indexes of TP, VLF, LF, SD1/SD2, and inflammatory cytokines and immune-regulating lymphocyte percentage (see Table 3).

## 4. Discussion

The MSS score and mortality of septic rats were significantly higher than those of the sham group and control group. GTS-21 can reduce the MSS score of sepsis; on the contrary, MLA significantly increased it. Studies have shown that the morbid fatigue of rats reflected by the MSS is closely related to the activation of glial cells in the central nervous system and the increase of serum IL-1 and other cytokines [23, 24], suggesting that sepsis rats may have both central and peripheral high levels of inflammation.

The results of ELISA confirmed that the levels of cytokines such as TNF-*α*, IL-1*α*, IL-10, IL-6, HMGB1, and sCD14 in septic rats increased significantly, indicating that the intrinsic immunity and adaptive immunity were activated; proinflammation and anti-inflammation coexisted in the early stage of sepsis [25]; CD4+CD25+Treg and TH17 simultaneously increased, and the ratio of Treg/TH17 decreased also confirmed that immune activation and suppression were accompanied by each other, but immune activation dominated at the early stage of sepsis.

Immune cells such as neutrophils and lymphocytes are innervated and regulated by the autonomic nerve according to the needs of the body's immunity [26]; sympathetic nerves have little effect on the number of lymphocytes [27], so the early inflammatory storm of sepsis should involve with the dysfunction of the autonomic nerve system, especially the vagus nerve. Therefore, CAP, as a key means of regulating the inflammation and immunity by the vagus nerve [28], plays a very important role of regulating inflammation and lymphocytes through the action on *α*7nAChR receptors by acetylcholine released from its terminals. This study also convincingly testified this law through modifying the septic systemic inflammation and immunity intensity by means of the specific *α*7nAChR agonists and antagonists. In this study, GTS-21 significantly inhibited hyperactive inflammation and immunity, while MLA was the opposite, indicating that CAP is a powerful negative regulator on inflammation and immunity. Here, we cannot help but think a problem: Does the overactivated inflammation stem from CAP's inhibition induced by sepsis?

Belonging to the vagus nerve, CAP's regulatory activity should be reflected by some indexes of HRV. Previous studies have confirmed that SDNN reflects both sympathetic and vagal activities and can be represented as total HRV changes [29]; HF and RMSSD represent vagal activity which is related to rapid changes in HRV [30]. In this study, SDNN, HF, and RMSSD all decreased in the sepsis group, indicating the inactiveness of the vagus nerve; the nonlinear indicators of SD1 and SD2 were significantly reduced simultaneously in the sepsis group, indicating that the two adjacent RR intervals have little variation and the scatter plot is very centralized, which illuminates the higher sympathetic excitement and lower vagal tone.

Through correlation analysis, SDNN, HF, RMSSD, SD1, and SD2 of HRV have strong correlations with MSS, inflammation, and immune indicators, while these indicators are strongly controlled by CAP as mentioned above; therefore, it can be concluded that SDNN, HF, RMSSD, SD1, and SD2 can be used to delineate the regulatory activity of CAP. The significant decrease of these indicators in the sepsis group directly suggests that CAP function is inhibited in sepsis.

Studies confirmed [31, 32] that stimulation of carotid sinus nerves and other vagus nerve endings in sepsis rats can evidently reduce the inflammation intensity in the brain; Pavlov et al. showed [33] that cholinesterase inhibitor which can pass through the blood-brain barrier (BBB) such as galantamine or Huperzine A can significantly reduce both plasma proinflammatory cytokine levels and mortality in septic rats, but cholinesterase inhibitors which cannot cross BBB have no such effect; what is more, the improvement effects of galantamine or Huperzine A were cancelled by means of CAP transection.

These two studies convincingly confirmed that CAP is a key and efficient means for the body to regulate the inflammation intensity in sepsis. The critical point of inflammation and immune disorders in sepsis should be focused on the central nervous system.

A study has confirmed [34] that MVZ is not only an important regulatory center responding to stress, but also an important integrated center of autonomic nerve and immune regulation. It controls the intensity of systemic inflammation dynamically through regulating the output of CAP [31, 35] under normal conditions and local inflammation. In sepsis, the suppressed CAP should be blamed for the MVZ's dysfunction and cause uncontrolled systemic inflammation. As we all know, the sensory fibers of the vagus nerve mainly enter the Nucleus Solitary Tract (NST), and the motor fibers are emitted from the Dorsal Vagus Motor Nucleus (DVMN). These structures all belong to MVZ. Therefore, the MVZ pathological changes should be related to CAP's suppression in the early stage of sepsis.

Cumulative studies have suggested that sepsis induce MVZ neuroinflammation [36–38]. It causes a surge of activated microglia [39], which results in inactiveness or even loss of neurons [40], and eventually, disorders of various autonomic functions appear [41, 42]. In the brain, inflammation exists mainly in the brain regions associated with autonomic nerves during sepsis; it suggests that sepsis selectively activates the inflammation of the autonomic regulatory center such as MVZ, which underlies the dysfunction of inflammation regulation [43]. In the intensive care unit, once a patient is diagnosed with septic encephalopathy, the possibility of death will rise dramatically [44]. It deduces that the selective inflammatory damage to the specific brain zone in the early stage of sepsis contributes to a poor prognosis. However, to my knowledge, by far, there is no relevant report on the pathological changes of MVZ and its effect on CAP in early sepsis. Therefore, it is very attractive question deserve to explore. MVZ consists of various types of neurons such as cholinergic neurons, catecholaminergic neurons, peptide energetic neurons, glutamate energetic neurons, and gamma-aminobutyric acid (GABA) energetic neurons, which are all involved in autonomic regulation, especially cholinergic neurons, catecholaminergic neurons [45]. In our study, immunofluorescence images showed that cholinergic neurons were mainly concentrated in the NST, DVMN, and Ventrolateral Reticular Nucleus (VLRN); catecholaminergic neurons were mainly distributed in NST, DVMN, VLRN, and the Area Postrema (AP). TUNEL showed that significant apoptosis occurs in MVZ cells in sepsis rats, and immunofluorescence colocalization suggested that sepsis caused evident apoptosis in MVZ cholinergic and catecholaminergic neurons. What is more, the higher the intensity of systemic inflammation (MLA group>model group>GTS-21 group), the more severe the apoptosis and the lower the expression of TH and CHAT. Correlation analysis between TH, CHAT, Caspase3 expression, and HRV suggests that the more severe the cholinergic and catecholaminergic neuron apoptosis, or the lower the TH and CHAT expression, the more inhibitory the CAP function. The upregulated expression of CHAT in MVZ indicates that the inflammatory information of peripheral organs is transmitted to MVZ through the vagus nerve which activates the cholinergic neurons in order to enhance the anti-inflammatory effect by CAP [46]. Therefore, the apoptosis or low expression of CHAT of cholinergic neurons in sepsis means an inadequate perception of inflammation and downregulation of anti-inflammation by MVZ and CAP, which in turn overactivate immune cells and eventually accelerate inflammation storm. In this study, although the cholinergic neurons in the model group are significantly apoptotic, they also significantly activated by inflammation in sepsis, so the expression of CHAT did not significantly decrease in the model group; there was a tendency towards an increase in the GTS-21 group which was related to the reduction of systemic inflammation, apoptosis, and the increasing activity of cholinergic neurons in MVZ. In the MLA group, accompanied by the aggravation of inflammation, the apoptosis of CHAT neurons increased further and the expression of CHAT decreased significantly when compared to the GTS-21 group.

Catecholaminergic neurons in MVZ respond sensitively to dangerous stress such as gastrointestinal toxicant [47], myocardial ischemia, and subarachnoid hemorrhage [48]. They project onto the DVMN and the medulla oblongata, by which they participate in the regulation of autonomic nerves to regulate inflammation and immunity [49]. The decrease in number and activity of catecholaminergic neurons will inevitably lead to insufficient activation of the DVMN and then reduce the output of CAP, which denotes that the curbing effect on inflammation by CAP decreases. The inflammation of the central nervous system not only enhances the TH expression but also promote the apoptosis of catecholaminergic neurons [50]; severe apoptosis of catecholaminergic neurons in sepsis gives rise to the reduction of CAP output. In this study, the expression of TH in MVZ catecholaminergic neurons decreased significantly in the sham group and the sepsis groups, indicating that catecholaminergic neurons were sensitive to stress, even surgical stress can also cause their dysfunction; in sepsis, sharp downregulation of expression of TH may be related to its excessive apoptosis. GTS-21 significantly reduced its apoptosis and increased the TH expression. MLA was completely the opposite. Although there are different levels of systemic inflammation in the model group, the GTS-21 group, and the MLA group, there is no significant difference in the CAP activity indicators among the three groups, probably because on the one hand, HRV is dominated by both sympathetic and vagal nerves; on the other hand, the terminal intervention of CAP has limited impact on HRV. Further researches such as exploring the relation between CAP and sympathetic nerves will be needed to clarify these queries.

## 5. Conclusions

Cholinergic and catecholaminergic neurons in MVZ manifest obvious apoptosis and low activity in sepsis, which leads to the inhibition of the CAP function and underlies the uncontrolled inflammation in early sepsis. Systemic inflammation storm has a significant effect on MVZ neuronal activity and apoptosis in sepsis. For the first time, it was confirmed that some indicators in HRV such as SDNN, HF band, RMSSD, SD1, and SD2 can reflect CAP regulatory activity, but they were hardly affected by the intervention of CAP.

## Figures and Tables

**Table 1:** Murine Sepsis Score.

Variable	Score and description
Appearance	0—coat is smooth
	1—patches of hair piloerected
	2—majority of the back is piloerected
	3—piloerection may or may not be present; the mouse appears “puffy”
	4—piloerection may or may not be present; the mouse appears emaciated

Level of consciousness	0—the mouse is active
	1—the mouse is active but avoids standing upright
	2—mouse activity is noticeably slowed. The mouse is still ambulant
	3—activity is impaired. The mouse only moves when provoked; movements have a tremor
	4—activity severely impaired. Mouse remains stationary when provoked, with possible tremor

Activity	0—normal amount of activity. The mouse is any of the following: eating, drinking, climbing, running, or fighting
	1—slightly suppressed activity. The mouse is moving around the bottom of the cage
	2—suppressed activity. The mouse is stationary with occasional investigative movements
	3—no activity. The mouse is stationary
	4—no activity. The mouse is experiencing tremors, particularly in the hind legs

Response to stimulus	0—the mouse responds immediately to auditory stimulus or touch
	1—slow or no response to auditory stimulus; strong response to touch (moves to escape)
	2—no response to auditory stimulus; moderate response to touch (moves a few steps)
	3—no response to auditory stimulus; mild response to touch (no locomotion)
	4—no response to auditory stimulus. Little or no response to touch. Cannot right itself if pushed over

Eyes	0—open
	1—eyes not fully open, possibly with secretions
	2—eyes at least half closed, possibly with secretions
	3—eyes half closed or more, possibly with secretions
	4—eyes closed or milky

Respiration rate	0—normal, rapid mouse respiration
	1—slightly decreased respiration (rate not quantifiable by eye)
	2—moderately reduced respiration (rate at the upper range of quantifying by eye)
	3—severely reduced respiration (rate easily countable by eye, 0.5 s between breaths)
	4—extremely reduced respiration (>1 s between breaths)

Respiration quality	0—normal
	1—brief periods of laboured breathing
	2—laboured, no gasping
	3—laboured with intermittent gasps
	4—gasping

**Table 2:** Time and frequency domain and nonlinear analysis of HRV among different groups.

	Control group	Sham group	Model group	GTS-21 group	MLA group
SDNN (ms)	3.96 ± 0.89	4.08 ± 0.90	1.59 ± 0.83^▲^	1.93 V ± 0.74^▲^	1.89 ± 0.72^▲^
RMSSD (ms)	4.25 ± 1.75	4.34 ± 1.26	1.69 ± 1.00^▲^	2.20 ± 0.96^▲^	2.32 ± 1.03^▲^
TP (ms^2^)	1213.27 ± 1022.20	2101.61 ± 345.48	1213.48 ± 818.07	1401.31 ± 678.42	1572.39 ± 747.65
VLF (ms^2^)	957.12 ± 978.45	1820.08 ± 368.32	992.03 ± 808.40	1200.65 ± 660.70	1370.90 ± 677.89
LF (ms^2^)	213.65 ± 109.33	232.95 ± 83.31	190.67 ± 25.46	170.85 ± 30.96	167.81 ± 74.43
HF (ms^2^)	42.56 ± 17.63	48.56 ± 13.06	30.75 ± 5.82^▲^	29.83 ± 6.44^▲^	33.67 ± 11.17^▲^
LF/HF (ms^2^)	5.20 ± 2.12	4.79 ± 1.01	6.45 ± 1.64	5.99 ± 1.66	4.75 ± 2.06
SD1 (ms)	3.00 ± 1.23	3.07 ± 0.89	1.19 ± 0.71^▲^	1.56 ± 0.68^▲^	1.64 ± 0.73^▲^
SD2 (ms)	4.64 ± 1.04	4.78 ± 1.42	1.87 ± 1.01^▲^	2.20 ± 0.92^▲^	2.08 ± 0.77^▲^
SD1/SD2	0.66 ± 0.27	0.69 ± 0.27	0.67 ± 0.27	0.74 ± 0.24	0.78 ± 0.22

HRV indexes including SDNN, RMSSD, HF, SD1, and SD2 of the model group, GTS-21 group, and MLA group were much higher than those of the control group. For SDNN, the *P* value and 95% CI were, respectively, 0.000, 1.359~3.380; 0.000, 1.022~3.043; and 0.000, 1.066~3.087. For RMSSD, they were 0.000, 1.070~4.054; 0.003, 0.559~3.543; and 0.005, 0.439~3.423. For HF, they were 0.006, 3.929~31.683; 0.003, 4.845~32.599; and 0.030, 1.012~8.766. For SD1, they were 0.000, 0752~2.864; 0.003,.0390~2.503; and 0.006, 0.304~2.417. For SD2, they were 0.000, 1.486~4.052; 0.000, 1.151~3.717; and 0.000, 1.271~3.837. There were no differences among the groups in such indexes as TP, VLF, LF, LF/HF, and SD1/SD2. SDNN: the standard deviation of all NN intervals; RMSSD: the root mean square successive difference; TP: total power; VLF: very low-frequency; LF: low frequency; HF: high frequency; SD1: standard deviation of width of a scatterplot of successive pairs of RR intervals; SD2: standard deviation of length of a scatterplot of successive pairs of RR intervals. ^▲^*P* < 0.05 vs. control group.

**Table 3:** Correlation analysis between HRV indexes and MSS, inflammatory cytokines, immune regulation, expression of CHAT, and TH/Caspase3.

	SDNN	RMSSD	TP	VLF	LF	HF	LF/HF	SD1	SD2
MSS	-0.883^∗∗^	-0.733^∗∗^	-0.196	-0.153	-0.42	-0.613^∗^	0.287	-0.732^∗∗^	-0.872^∗∗^
TNF-*α*	-0.798^∗∗^	-0.712^∗∗^	0.16	0.2	0.21	-0.429^∗^	0.25	-0.711^∗∗^	-0.756^∗∗^
IL-1*α*	-0.786^∗∗^	-0.707^∗∗^	0.1	0.12	0.14	-0.401^∗^	0.28	-0.706^∗∗^	-0.747^∗∗^
IL-6	-0.813^∗∗^	-0.699^∗∗^	0.08	0.11	0.19	-0.415^∗^	0.23	-0.698^∗∗^	-0.787^∗∗^
IL-10	-0.650^∗∗^	-0.607^∗∗^	0.01	0.04	0.19	-0.425^∗^	0.23	-0.607^∗∗^	-0.609^∗∗^
sCD14	-0.632^∗∗^	-0.575^∗∗^	0.18	0.2	0.07	-0.407^∗^	0.362^∗^	-0.574^∗∗^	-0.596^∗∗^
HMGB1	-0.747^∗∗^	-0.687^∗∗^	0.17	0.19	0.1	0.36	0.26	-0.687^∗∗^	-0.704^∗∗^
Treg	-0.820^∗∗^	-0.727^∗∗^	0.09	0.06	0.47	0.01	0.27	-0.726^∗∗^	-0.816^∗∗^
TH17	-0.683^∗∗^	-0.692^∗∗^	0.11	0.09	0.42	0.05	0.18	-0.691^∗∗^	-0.662^∗∗^
Treg/TH17	0.579^∗^	0.671^∗∗^	0.15	0.12	0.45	0.08	0.18	0.670^∗∗^	0.549^∗^
CHAT	0.406^∗∗^	0.333^∗^	0.08	0.052	0.261	0.269	0.046	0.332^∗^	0.400^∗∗^
TH	0.570^∗∗^	0.478^∗∗^	-0.07	-0.091	0.139	0.245	-0.021	0.477^∗∗^	0.570^∗∗^
Caspase3	-0.675^∗∗^	-0.564^∗∗^	-0.187	-0.159	-0.281	-0.411^∗∗^	-0.008	-0.564^∗∗^	-0.668^∗∗^

^∗∗^ *P* < 0.01, ^∗^*P* < 0.05.

## Data Availability

Because this study is supported by the Guizhou Provincial Science and Technology Foundation, the data will not make known to public until the research project is checked and accepted. Therefore, we declare that all the datasets used and/or analysed during the current study are available from the author on reasonable request. The link email is mrbright789@sina.com.
